# “POLAR-izing” Findings From Trials of Neuroprotection for Oxaliplatin Neuropathy

**DOI:** 10.1093/jncics/pkac076

**Published:** 2022-10-29

**Authors:** Christina Teng, Janette L Vardy

**Affiliations:** Faculty of Medicine and Health, University of Sydney, Sydney, Australia; Concord Cancer Centre, Concord Repatriation General Hospital, Sydney, Australia; Faculty of Medicine and Health, University of Sydney, Sydney, Australia; Concord Cancer Centre, Concord Repatriation General Hospital, Sydney, Australia

Chemotherapy-induced peripheral neuropathy (CIPN) is a major issue for many cancer patients receiving chemotherapy agents such as platinums, taxanes, and vinca alkaloids, leading to dose reductions or early cessation of treatment (1) and interfering with quality of life and daily function (2). For platinum drugs, it is predominantly a sensory neuropathy, characterized by numbness and tingling, temperature sensitivity, and discomfort, or less commonly pain, in the hands and feet (3). Our recent systematic review in patients treated with adjuvant oxaliplatin for colorectal cancer (CRC) found CIPN affected 58% of patients 6 months after completion of chemotherapy, 45% of patients at 12 months, and 24% at 36 months (4). However, the incidence of CIPN in routine clinical practice is likely higher than reported in clinical trials, with 73% of our real-world cohort reporting at least moderate neuropathy symptoms 6 months after completion of adjuvant oxaliplatin for CRC, with 80% requiring a dose modification or early cessation, most commonly because of neuropathy (5).

The putative mechanism for CIPN due to platinum chemotherapy is believed to be oxidation injury, secondary to mitochondrial damage (6). Other than reducing exposure to the chemotherapy agent with treatment delays, dose reduction, or cessation, which can compromise treatment efficacy, evidence for prevention of CIPN is limited (7). Timing for early discontinuation of platinum drugs is particularly challenging as they can cause a “coasting” effect, with CIPN continuing to increase for the first 3 months after ceasing treatment (8). There are minimal options for treatment once the nerve damage has occurred. The American Society of Clinical Oncology guidelines recommend duloxetine for treatment of painful CIPN, and there is limited evidence that exercise may help (7).

We commend Pfeiffer and colleagues (9) for 2 well-designed phase III multicenter, randomized, double-blind, placebo-controlled trials evaluating calmangafodipir (CaM) for the prevention of CIPN secondary to oxaliplatin for CRC: one in the adjuvant setting (Preventive Treatment of OxaLiplatin Induced peripherAl neuRopathy [POLAR-A]) and one for metastatic CRC (POLAR-M). CaM, an iron chelator, is thought to act as a neuroprotectant by attenuating the formation of cellular oxidative stress (10). In mouse models, CaM was found to prevent small-fiber neuropathy from oxaliplatin (11).

POLAR-A and POLAR-M were terminated early by the data safety monitoring board because of hypersensitivity reactions in the CaM-treated participants (12 of 336 CaM vs 2 of 246 placebo). This resulted in smaller than planned sample sizes with no participant completing either study according to the protocol and a lack of longer-term efficacy or safety data. To optimize the statistical power of the analyses, data from the 2 studies were pooled, and the authors used a modified intention-to-treat analysis that included 88% of the 434 participants randomly assigned, with data for the primary endpoint for 351 participants. In POLAR-A, participants were randomly assigned to intravenous CaM 5 μmol/kg (n = 120) or placebo (n = 119), and in POLAR-M, to CaM 2 μmol/kg (n = 54), CaM 5 μmol/kg (n = 55), or placebo (n = 57), but only 29%-38% received 12 cycles of study treatment. The trial findings did not indicate a beneficial effect of CaM on the primary endpoint of CIPN at 9 months after starting oxaliplatin, using the first 4 items of the patient-reported Functional Assessment of Cancer Therapy–Gynecologic Oncology Group–Neurotoxicity subscale questionnaire. Furthermore, moderate-to-severe CIPN at 9 months was greater in the participants receiving CaM 5 μmol/kg compared with placebo (54% vs 40%: relative risk [RR] = 1.37; *P* = .045).

The POLAR studies were informed by PLIANT, a phase I dose finding study (n = 11) and phase II trial in 173 participants with metastatic CRC randomly assigned to receive placebo (n = 60) or CaM at 2 (n = 57), 5 (n = 45), or 10 (n = 11) μmol/kg. (The 10 μmol/kg dose was reduced to 5 μmol because of greater non-neurotoxic grade 3 adverse events) (10). The PLIANT study was negative for the primary endpoint of grade 2 or greater physician-assessed neuropathy after 8 cycles of folinic acid, 5-fluorouracil, and oxaliplatin. Analysis of secondary endpoints showed less cold allodynia and sensory symptoms during chemotherapy in CaM-treated participants. Assessments at 3 and 6 months after oxaliplatin reported more sensory symptoms in the placebo group than CaM-treated participants. Comparison between the studies is challenging because of differences in primary endpoints and time points, CIPN measurement instruments, and cumulative doses of oxaliplatin.

Of note, there was no hypersensitivity reported in any participants in the PLIANT study (10). Hypersensitivity, described as flushing, lightheadedness, or dizziness, was however reported in 35 of 40 patients in the first human cohort to receive manganese-based contrast (Manganese dipyridoxyl diphosphate), from which CaM was developed, for hepatobiliary imaging (12). Flushing is commonly experienced with computed tomography contrast and quite different to true hypersensitivity, characterized by hypoxemia or hemodynamic instability. It is unclear how hypersensitivity was defined in the POLAR studies.

In POLAR, the proportion of participants receiving 12 cycles of oxaliplatin was reported, but the mean or median number of oxaliplatin cycles given was not provided (9). The relative risk for the primary endpoint was adjusted for the cumulative dose of oxaliplatin, but it is also important for the authors to report dose delays or discontinuation of oxaliplatin and the reasons for these, as this commonly occurs in clinical practice and could potentially influence the findings. Compare 2 participants for example. For participant A, who received placebo with chemotherapy, the physician dose-reduced oxaliplatin after cycle 1 because of unacceptable neuropathy then ceased it after cycle 3; so, we might expect improvement in the participant’s CIPN by month 9 when the primary endpoint is evaluated. In contrast, participant B receives CaM and has attenuated CIPN symptoms, enabling the participant to complete 6 months of oxaliplatin before getting moderate neuropathy and is then evaluated at month 9. Both may report the same CIPN symptoms, but we may have “missed” any beneficial impact of CaM. This also may explain the trend for increased cumulative oxaliplatin dose seen in the CaM recipients.

Also notable, POLAR participants who stop oxaliplatin and/or study therapy essentially stop having fortnightly CIPN assessments recorded for study purposes. We know oxaliplatin neuropathy can worsen after cessation of treatment, and this is reflected in the symptom graphs (Supplementary Figure 1 and 2, available online [9]), with increases in the symptom scores between cycle 12 and month 9. It is possible that if CaM attenuates symptoms during the treatment, there might be ongoing oxidative stress, reactive oxygen species, and nerve damage even after CaM is ceased, leading to coasting or rebound symptoms. This raises the question of whether it is sufficient to give CaM, or any other neuroprotective agent, concurrently with oxaliplatin, or if we need to continue it beyond chemotherapy cessation to get the desired benefit.

Pfeiffer et al. (9) propose White participants might benefit more from CaM and reported Asian participants were more likely to report worse CIPN. We recommend caution in drawing these conclusions, as it is based on a small, unplanned sample of 57 Asian participants, and the findings were seen only in the metastatic setting and were not statistically significant. Although differences by race and ethnicity have been observed in large datasets assessing other toxicities, there is no conclusive evidence that Asian patients have more CIPN than patients (13,14). Conversely, pooled data from 6 folinic acid, 5-fluorouracil, and oxaliplatin studies suggested Asian populations might be less susceptible to oxaliplatin neurotoxicity (15).

Overall, this was a negative study where people randomly assigned to the active treatment had more CIPN but also received more oxaliplatin. Clearly, CaM is not ready for “prime time” due to highlighted safety concerns combined with the perceived lack of benefit and potentially more neurotoxicity. There were challenges evaluating the efficacy of the therapy, meaning that a benefit from CaM, if it existed, might not have been identified. We concur with Pfeiffer et al. (9) that the results, like other interventional studies aimed at preventing CIPN, highlight the difficulties of designing CIPN prevention studies. We need to continue to strive toward clinically meaningful ways to measure effectiveness of the active treatment to incorporate into trial design (16), while considering the potential cost-benefit to the patient of early cessation or dose reductions in potentially life-saving chemotherapy (17).

## Funding

No funding was used for the writing of this editorial.

## Notes

**Role of the funder:** Not applicable.

**Disclosures**: JV and CT have no disclosures to declare.

**Author contributions:** JV: conceptualization, writing—original draft, writing—review & editing. CT: conceptualization, writing—original draft, writing—review & editing.

## Data Availability

No new data were generated or analyzed for this editorial.
